# Two‐year outcomes of epicranial focal cortex stimulation in pharmacoresistant focal epilepsy

**DOI:** 10.1111/epi.18448

**Published:** 2025-05-16

**Authors:** Andreas Schulze‐Bonhage, Martin Hirsch, Susanne Knake, Ann Mertens, Michael Rademacher, Elisabeth Kaufmann, Josua Kegele, Carolin Jenkner, Volker Coenen, Martin Glaser, Sergiu Groppa, Yaroslav Winter, for the EASEE Study Group

**Affiliations:** ^1^ Epilepsy Center, University Medical Center University of Freiburg Freiburg Germany; ^2^ NeuroModul Basic University of Freiburg Freiburg Germany; ^3^ Bernstein Center of Computational Neuroscience University of Freiburg Freiburg Germany; ^4^ European Reference Network EpiCARE Lyon France; ^5^ Neurology University Hospital Marburg Marburg Germany; ^6^ Neurology University Hospital Gent Ghent Belgium; ^7^ Epileptology University Hospital Bonn Bonn Germany; ^8^ Department of Neurology, University Hospital Ludwig Maximilian University Munich Germany; ^9^ Department of Neurology and Epileptology, Hertie Institute for Clinical Brain Research University of Tübingen Tübingen Germany; ^10^ Clinical Trials Unit, University Medical Center University of Freiburg Freiburg Germany; ^11^ Department of Stereotactic and Functional Neurosurgery, University Medical Center University of Freiburg Freiburg Germany; ^12^ Department of Neurosurgery University of Mainz Mainz Germany; ^13^ Department of Neurology, Saarland University Medical Center University of Saarland Homburg Germany

**Keywords:** epileptic focus, focal epilepsy, long‐term outcome, neuromodulation, neurostimulation

## Abstract

**Objective:**

This study was undertaken to report on the long‐term safety and efficacy of epicranial focal cortex stimulation (FCS) using the EASEE device as adjunctive neuromodulatory therapy in improving seizure control in adults with pharmacoresistant epilepsy originating from one predominant epileptogenic zone.

**Methods:**

Prospective open‐label follow‐up of patients from the EASEE II and PIMIDES I clinical trials was done for a period of 2 years after the epicranial implantation of the EASEE electrode and stimulator device.

**Results:**

Thirty‐three patients underwent device implantation, and stimulation was activated in 32 patients. Of these, 26 patients continued stimulation up to 2‐year follow‐up and provided seizure diary data for efficacy analysis. The 50% responder rate at 2‐year follow‐up was 65.4% (95% confidence interval = 44.3–82.8), corresponding to a median seizure frequency reduction of 68%. Patients reported improved health‐related quality of life. Tolerability was excellent, and there were no severe adverse events considered to be related to implantation or stimulation, nor were adverse effects on mood or cognition reported.

**Significance:**

Results of the 2‐year follow‐up show that epicranial FCS is well tolerated by patients while providing improved seizure control in the long term. It thus offers a minimally invasive treatment option for patients with a predominant epileptic focus.

**Key points**

- More than 80% of patients chose to continue treatment with epicranial focal cortex stimulation for a 2‐year period.
- Focal cortex stimulation proved safe over 2 years of treatment, without any serious adverse event reported related to either implantation or stimulation.
- Long‐term stimulation of the epileptic focus using the EASEE device continued to be effective.
- A 68% median seizure reduction and a responder rate of 65% was achieved after 2 years.

## 1 INTRODUCTION

More than one third of patients with focal epilepsy suffer from pharmacoresistant seizures, and only a subgroup of these patients are appropriate candidates for epilepsy surgery. Neurostimulation approaches can supplement the treatment armamentarium for patients suffering from ongoing seizures despite optimized pharmacotherapy. Neurostimulation can target and modulate widespread networks, as with vagus nerve stimulation (VNS) or deep brain stimulation (DBS) of thalamic nuclei, or directly affect the epileptogenic zone where seizures are generated.^1^, ^2^ Recently, epicranial focal cortex stimulation (FCS) has been approved for the treatment of pharmacoresistant focal epilepsy, having shown efficacy in reducing seizures in a combined 6‐month outcome analysis of two prospective, open‐label single‐arm clinical trials (Epicranial Application of Stimulation Electrodes for Epilepsy (EASEE II) Responder rates, defined as at least 50%/75%/90% reduction in seizure rate and Patient Individualized Modulationand Intervention by Directed Epicranial Stimulation (PIMIDES I)).^3^, ^4^

FCS is applied over the individual epileptogenic brain region as determined by clinical data, including magnetic resonance imaging (MRI), electroencephalographic (EEG) recordings, and seizure semiology. FCS uses a combined neurostimulation approach consisting of high‐frequency burst stimulation distributed over the day and direct current (DC)‐like cathodal stimulation applied for a period of 20 min per day. Additionally, patients who experience a focal aware phase of their seizures can trigger ictal stimulation via a handheld device.^5^

Evidence for the efficacy of neurostimulation of the epileptic focus is based on research in experimental models of epilepsy,^6^, ^7^ on modeling of direct current (DC) and alternating current (AC) stimulation effects on cortical dynamics,^8^ and on multiple studies on transcranial DC and AC stimulation in humans.^9^, ^10^, ^11^, ^12^ Furthermore, the epicranial placement of the stimulation electrode has been shown to enhance stimulation effects by a factor of four compared to transcutaneous stimulation.^13^ Reviews of the concepts underlying focal cortex stimulation have been provided by Schulze‐Bonhage et al.^2^, ^3^, ^4^ Over the initial treatment period of 6 months, more than half of the study patients undergoing FCS were considered responders based on a reduction of their seizure frequency by at least 50% compared to baseline. During this treatment period, only mild adverse effects were reported that were considered to be related to the implantation, mostly reflecting local skin irritation at the site of electrode placement; there were no adverse effects reported related to the stimulation itself.^3^, ^4^ Here, we report study results from long‐term follow‐up of these two clinical trials over a 2‐year postimplantation period, descriptively analyzing treatment efficacy and tolerability.

## 2 MATERIALS AND METHODS

This is a pooled analysis of 2‐year follow‐up data from 2 prospective multicenter single‐arm trials (EASEE II and PIMIDES I), assessing safety and efficacy of neurostimulation using the EASEE System (Precisis GmbH), a CE‐certified medical device consisting of an epicranially implanted five‐contact electrode array connected to a programmable neurostimulator placed at the pectoral region in adult patients with pharmacoresistant focal epilepsy.

The 33 subjects who underwent surgery in the trials were 18–75 years of age and had focal onset seizures uncontrolled by at least two antiseizure medication (ASM; mean = 7.8, range = 3–15 prior ASMs) treatments (for further details, see Table 1); four patients had undergone prior VNS. Etiologies were heterogeneous, with the most frequent underlying MRI‐based suspected etiology being focal cortical dysplasia; 10 patients were nonlesional on MRI. Included in the study were adult patients with focal onset seizures uncontrolled by at least two ASM treatments, a seizure frequency of ≥3/month, temporolateral or extratemporal seizure origin, a distinct, stereotyped, and countable seizure semiology, and a predominant epileptic focus area from which the majority of seizures were considered to arise; neither an MRI lesion nor invasive recordings were required for focus localization. In the PIMIDES subgroup, patients had to have one seizure type with an initial aware phase. The complete list of inclusion and exclusion criteria is provided as File S1.

**TABLE 1:** Demographics of patients included in trial who underwent surgery.

Characteristic	Value	Total
Participants, *N*		33
Sex, *n* (%)	Male: 18 (54%) Female: 15 (46%)	
Age, years, mean ± SD, range	34.6 ± 13.5, 18–75	
Duration of epilepsy, years, mean ± SD, range	20.4 ± 12.4, 3.2–66.2	
Monthly seizure frequency at baseline, mean/median	33.7/12	
Focus localization, *n*		
Temporal	Left: 11 Right: 4	15
Frontal	Left: 4 Right: 5	9
Other	Left: 5 Right: 4	9
Focus lateralization, *n* (%)	Left: 20 (61%) Right: 13 (39%)	33 (100%)
ASM pretreatments, *n*, mean ± SD, range	7.8 ± 4.3, 3–15	

Abbreviation: ASM, antiseizure medication.

Seizures and seizure types (focal aware, focal unaware, and focal to bilateral tonic–clonic) were reported by patients in seizure diaries. The study protocol had been approved by ethics boards of the seven participating centers in Bonn, Freiburg, Mainz, Marburg, Munich, and Tübingen, Germany and Ghent, Belgium. The respective studies were registered in the German Clinical Trials Registry (DRKS) and their meta‐analysis under PROSPERO.

The epicranial electrode array consists of five stimulation electrodes in a pseudo‐Laplacian arrangement with a maximal interelectrode distance of 50 mm, connected to the stimulator placed subcutaneously in the pectoral region at the side of stimulation. Stimulation consisted of DC‐like stimulation (DLS) with cathodal pulses of up to 2‐mA intensity, applied via the central electrode for a period of 20 min per day, and high‐frequency stimulation (HFS) bursts of up to 4‐mA intensity, with rectangular biphasic symmetric pulses at 100 Hz, a pulse width of 160 μs, and trains of 500 ms applied every 2 min.^14^, ^15^ The 17 patients from the PIMIDES I cohort could additionally apply on‐demand patient‐controlled neurostimulation for 10–60 s according to chosen settings. This protocol was applied to all patients during the first 6 months of stimulation. In a subgroup of patients, the HFS was later changed to low‐frequency stimulation; seven patients had 1‐Hz stimulation, and one underwent 0.5‐Hz stimulation.

The 33 implanted patients (18 male, 15 female) undergoing stimulation had a mean age of 34.6 years (range = 18–75 years; see Table 1) and had foci across all brain regions, with the most frequent sites of seizure origin being temporal and frontal. In the long‐term follow‐up period, study visits were scheduled regularly (12, 16, and 24 months postimplantation) at the respective epilepsy centers.

Seizure frequencies as documented in seizure diaries were assessed on a monthly basis (30‐day intervals) following implantation. Therefore, the time intervals of the seizure documentation do not correspond with the scheduled study visits. For time intervals with partially missing documentation, the seizure rate was imputed based on the number of available days (number of seizures in the respective interval/number of available days) × 30. As a sensitivity analysis, the missing month was replaced by the patient's medium seizure frequency in the preceding 6 months. Seizure frequency reductions in comparison to the baseline month (as a percentage) are displayed descriptively in boxplots for all monthly intervals (Figure 2A).

Responder rates, defined as at least 50%/75%/90% reduction in seizure rate from baseline, were calculated for all patients with seizure diary data available (complete case) for stimulation month 12 (day 331–360), stimulation month 15 (day 421–450), and stimulation month 23 (day 661–690) and given with the corresponding 95% exact confidence intervals (CIs). Furthermore, two sensitivity analyses were performed, including the worst‐case intent‐to‐treat (ITT) approach of counting missing data as nonresponse, and a last observation carried forward (LOCF) approach, both including the nonstimulated patient as nonresponder. All analyses are reported descriptively, as the primary efficacy endpoint was already evaluated in stimulation month 6.^3^, ^4^

Safety analyses were performed on the safety set, including all patients for whom the surgery was performed. Adverse events (AEs) were coded with MedDRA and summarized by body system. The incidence of device/procedure‐related serious AEs (SAEs) for the surgical implant procedure and the following 4 months are summarized with corresponding exact one‐sided 95% CIs based on a binominal distribution. Any SAE occurring was reviewed by an independent data safety and monitoring board (DSMB).

At the 12‐, 18‐, and 24‐month follow‐up visits, patients were asked to complete the Quality of Life in Epilepsy–Problems questionnaire (QOLIE‐31‐P^16^, ^17^) and the Neurological Disorders Depression Inventory for Epilepsy (NDDI‐E^18^), and neurocognition was assessed using the EpiTrack screening tool (Eisai^19^). Statistical significance assessment of QoL, mood, cognition, and drug load as indexed by daily defined doses (DDD) in polytherapy was performed using paired *t*‐tests, with *p* <0 .05 uncorrected for multiple testing considered significant. For evaluation of mood changes, critical thresholds for NDDI‐E scores indicating reliable changes were based on the Reliable Change Index (RCI) according to Jacobson and Truax^20^ RCI (with 95% CI) was computed between the prospective baseline period and 24‐month follow‐up (or earlier if 24 months were not reached). Reliability and thresholds for reliable change were taken from Hohmann et al.^21^ Changes of the NDDI‐E total score were classified as either unchanged, improved, or deteriorated at follow‐up accordingly.

## 3 RESULTS

Thirty‐three patients underwent device implantation, and stimulation was activated in 32 patients. Overall, 26 of 32 (81.25%) patients undergoing stimulation continued epicranial FCS and provided seizure diary data for the analyzed period of 2 years, despite the need for generator replacement during the second year of stimulation.

**FIGURE 1:**
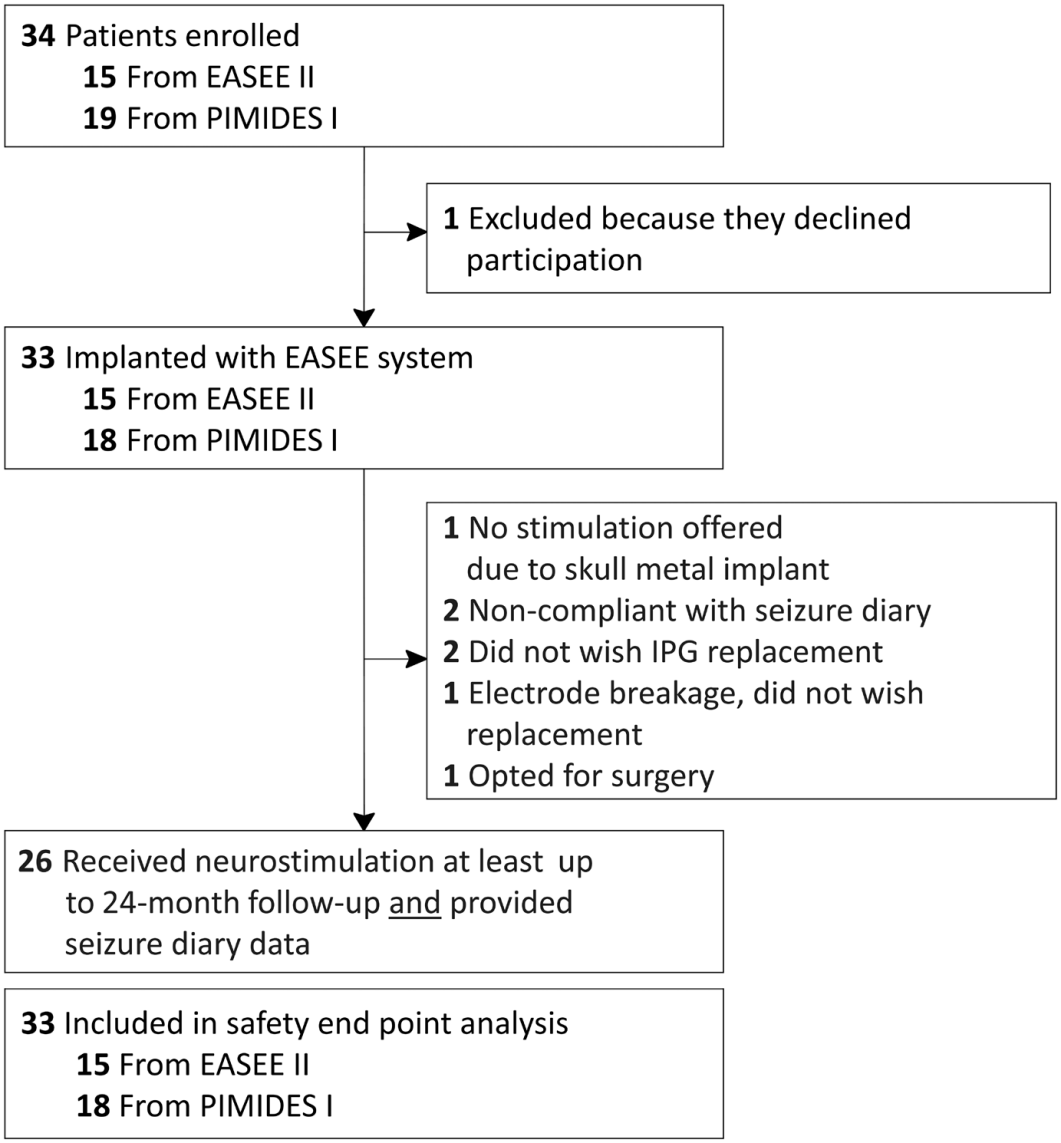
Study flow diagram. IPG, implantable pulse generator.

A CONSORT diagram with an overview of all enrolled patients and reasons for early terminations can be found in Figure 1. Of the 33 implanted patients, stimulation was not activated in one due to the presence of metal implants in the skull only detected during device implantation. Of the six patients who did not contribute 2‐year efficacy data, two were not sufficiently compliant with filling out their seizure diaries (one withdrew after the 8‐month follow‐up visit, whereas the other completed the 24‐month follow‐up visit); one patient with an electrode breakage resulting from a fall did not wish a device replacement 12 months after implantation; two patients opted against device replacement after 15 and 16 months, respectively (one of them despite showing a good response to treatment); and one patient considered efficacy insufficient and underwent epilepsy surgery in his eloquent motor area after 22 months of stimulation.

Up to the 2‐year follow‐up, a total of 55.8 device implant years was reached over all patients in whom stimulation was successfully activated, and the 26 patients continuing treatment had all undergone an EASEE Power replacement (after mean = 364 ± 36 days).

### 3.1 Safety

A total of 25 SAEs emerged in 13 patients during the 2‐year follow‐up period, corresponding to an incidence of 39.4% (95% CI = 22.9–57.9). This included two psychiatric SAEs (see below), abnormal laboratory findings like hyponatremia, and gastrointestinal, respiratory, and vascular symptomatology. There were no SAEs that were considered to be related to the device or stimulation (incidence = 0%, 95% CI = 0.0–10.6). An overview of the MedDRA‐coded SAEs is given in Table 2. There were no deaths observed during the 2‐year follow‐up.

**TABLE 2:** Reported percentage of patients with a given serious adverse event during the 24‐month period following device implantation (cutoff incidence > 5%).

Reported serious adverse events (MedDRA type)	Most frequent	Adverse events incidence	
		*n*	%
Total patients		33	100
Patients with at least one SAE		13	39.4
Nervous systems disorders		5	15.2
	Epilepsy	2	6.1
	Status epilepticus	2	6.1
Gastrointestinal disorders		3	9.1
Injury, poisoning, and procedural complications		3	9.1
	Toxicity to various agents	2	6.1
Psychiatric disorders		3	9.1
Vascular disorders		2	6.1

Abbreviation: SAE, serious adverse event.

Epilepsy‐related AEs were coded in cases of transient increases in seizure frequency/seizure clustering or seizure‐associated falls. As reported in a previous publication,^3^, ^4^ two patients (6.1%) experienced status epilepticus during the first 6 months of stimulation, one of them being the patient in whom stimulation was not initiated due to the presence of metal implants in the skull. No further cases of status epilepticus were reported during the 2‐year follow‐up; one patient reported a seizure cluster early in the stimulation period. One local infection of the device pocket was reported 3 days after a second generator replacement surgery, resolving without sequelae upon antibiotic treatment.

Overall, 166 AEs were reported in 27 patients over the 2‐year follow‐up period. Of these, 47 events were classified as adverse device effects (ADEs) and affected 18 patients. More than half (24/47) of the ADEs occurred within 1 month following implantation and were mostly related to the surgical procedure (e.g. postoperative pain, headache, hematoma); a further 15 of 47 (32%) events occurred within 6 months postimplantation and included epilepsy‐related events, tingling/paresthesia, and discomfort/pain at implant site. Only eight events were reported in the subsequent 18 months (including epilepsy‐related events and postoperative pain following Implantable pulse generator exchanges).

At the group level, NDDI‐E scores remained stable over the 2‐year follow‐up period (see Figure 4). On an individual level, most patients exhibited no reliable change in NDDI‐E scores. Five patients demonstrated clinically significant improvement, reporting reduced depressive symptoms, whereas one patient showed a deterioration based on the RCI after 2 years. Two SAEs related to depression led to hospitalization. Both individuals had elevated baseline NDDI‐E scores (17 and 18, respectively) and had repeatedly reported depressive symptoms, which were documented as AEs. In one case, depressive symptoms began prior to the initiation of stimulation. This patient was hospitalized at month 16 of treatment, with the event classified as an SAE; symptoms resolved within 1 month. The second patient was hospitalized in month 20, with depressive symptoms subsequently resolving. Neither of these AEs was deemed stimulation‐related by the treating principal investigator.

Assessment of neurocognition using EpiTrack showed a statistically significant yet minor improvement in total cognitive scores over time compared to baseline (see Figure 4); data were, however, not corrected for possible effects of repeated application. A single patient transiently complained of a mild memory deterioration after increasing the dosage of zonisamide from 200 to 300 mg/day (+100 mg/day); this was not considered stimulation‐related.

### 3.2 Efficacy

**FIGURE 2:**
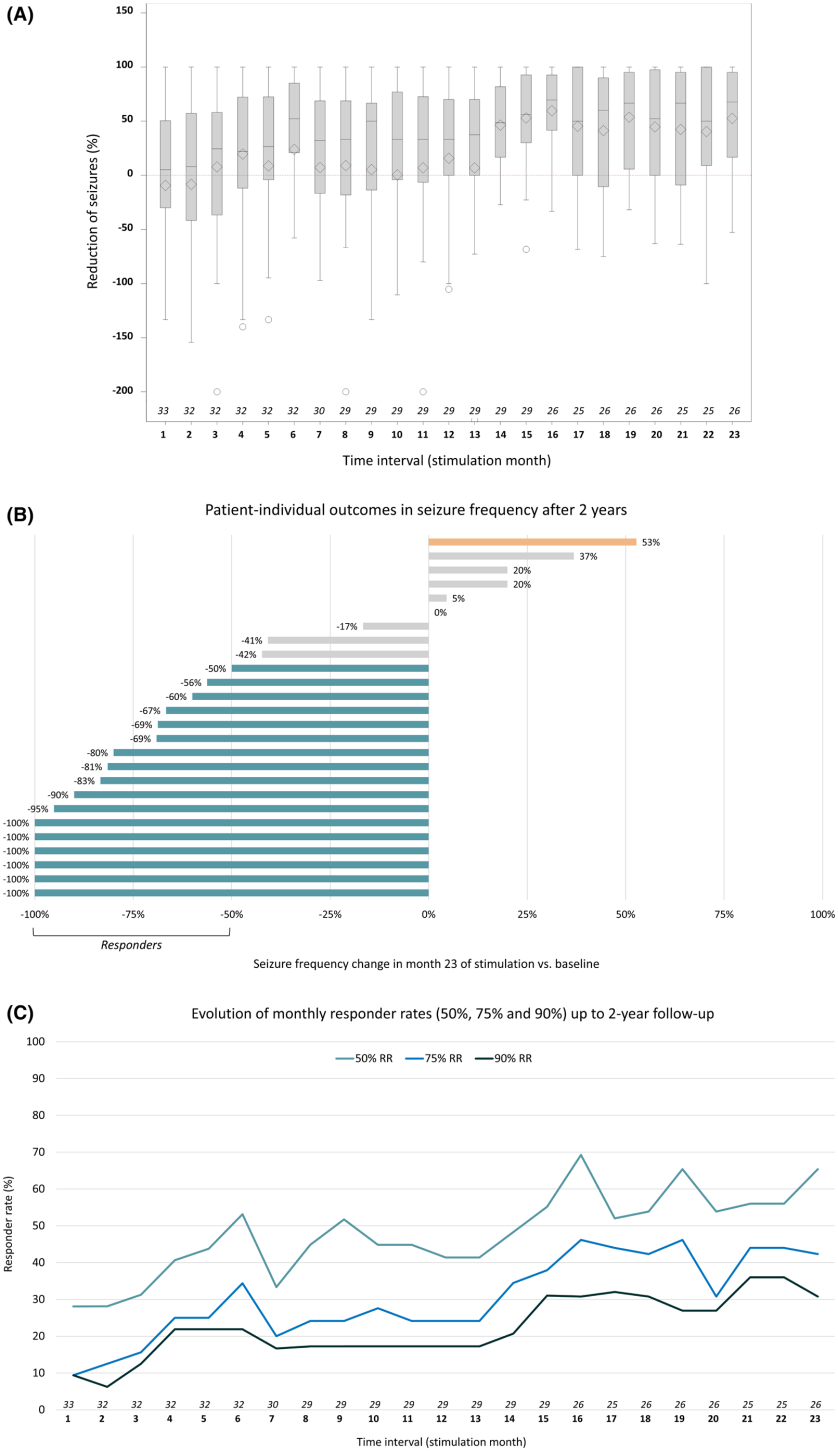
(A) Box plots with mean (diamond) and median (horizontal line in plot) seizure frequency reduction and spread for 2 years following device implantation given per stimulation month (stimulation was generally turned on 1 month after implantation). Note that months without reliable seizure counts in patients´ diaries are considered missing, as reflected by the number given for each month. (B) Patient‐individual outcomes at 2‐year follow‐up for the 26 patients with seizure diary data available (cf. panel A). Responders with at least 50% seizure reduction are depicted in blue, the one patient with >50% seizure increase in orange. (C) Development of treatment response over time (percentage of patients with 50%, 75%, and 90% seizure reduction) over time. RR, responder rate.

At the group level, the mean seizure frequency declined from 33.7 at baseline to 19.2 during the last month (stimulation month 23), and the median seizure frequency declined from 12 at baseline to 5. Figure 2A displays the mean and median seizure frequency reductions on a patient level. A declining trend was found from baseline to the end of the follow‐up period, with a median seizure frequency reduction of 56% in stimulation month 15 and 68% in stimulation month 23. Similarly, the responder rate increased to 55% in stimulation month 15 and 65% in the final month (see Table 3 and Figure 2C). During the last treatment month, 42% of patients had a seizure reduction of >75% and 31% of >90%, and 23% of patients reported no seizures, whereas one patient had an increase of >50% at last follow‐up (see Figure 2B,C).

**TABLE 3:** Responder rates, defined as at least 50% (75%, 90%) reduction in seizure rate from baseline, calculated for all patients with seizure diary data available (complete case).

Time interval	Complete case		Worst‐case approach		LOCF analysis	
	*n*	Percentage of responders, % [95% CI]	*n*	Percentage of responders, % [95% CI]	*n*	Percentage of responders, % [95% CI]
50% responders						
Stimulation month 12	29	41.4% [23.5–61.1]	33	36.4% [20.4–54.9]	33	45.5% [28.1–63.7]
Stimulation month 15	29	55.2% [35.7–73.6]	33	48.5% [30.8–66.5]	33	57.6% [39.2–74.5]
Stimulation month 23	26	65.4% [44.3–82.8]	33	51.5% [33.5–69.2]	33	66.7% [48.2–82.0]
75% responders						
Stimulation month 12	29	24.1% [10.3–43.5]	33	21.2% [9.0–38.9]	33	24.2% [11.1–42.3]
Stimulation month 15	29	37.9% [20.7–57.7]	33	33.3% [18.0–51.8]	33	36.4% [20.4–54.9]
Stimulation month 23	26	42.3% [23.4–63.1]	33	33.3% [18.0–51.8]	33	39.4% [22.9–57.9]
90% responders						
Stimulation month 12	29	17.2% [5.9–35.8]	33	15.2% [5.1–31.9]	33	18.2% [7.0–35.5]
Stimulation month 15	29	31.0% [15.3–50.8]	33	27.3% [13.3–45.5]	33	30.3% [15.6–48.7]
Stimulation month 23	26	30.8% [14.3–51.8]	33	24.2% [11.1–42.3]	33	27.3% [13.3–45.5]

*Note*: Furthermore, two sensitivity analyses were performed, including the most conservative approach of counting missing data as nonresponse in an intent‐to‐treat approach, which also considered the patient a nonresponder who was implanted but did not receive any stimulation due to the presence of metal implants. Furthermore, an LOCF analysis was performed, which considered the available last follow‐up data as constant for the further time course, with any nonresponder at last follow‐up counted as a nonresponder, again including the nonstimulated patient.

Abbreviations: CI, confidence interval; LOCF, last observation carried forward.

To account for the effects of missing data and for patients discontinuing participation in the trials, additional analyses were performed, which are reported in Table 3. A worst‐case scenario used an ITT approach, considering any missing data as nonresponse to treatment, and including the nonstimulated patient as a nonresponder. This approach yielded a responder rate of 52% in stimulation month 23. An LOCF approach considered the last available follow‐up response as permanent and resulted in a similar responder rate compared to the complete case (67% vs. 65%), suggesting that there was no major bias resulting from nonresponders stopping the treatment earlier.

**FIGURE 3:**
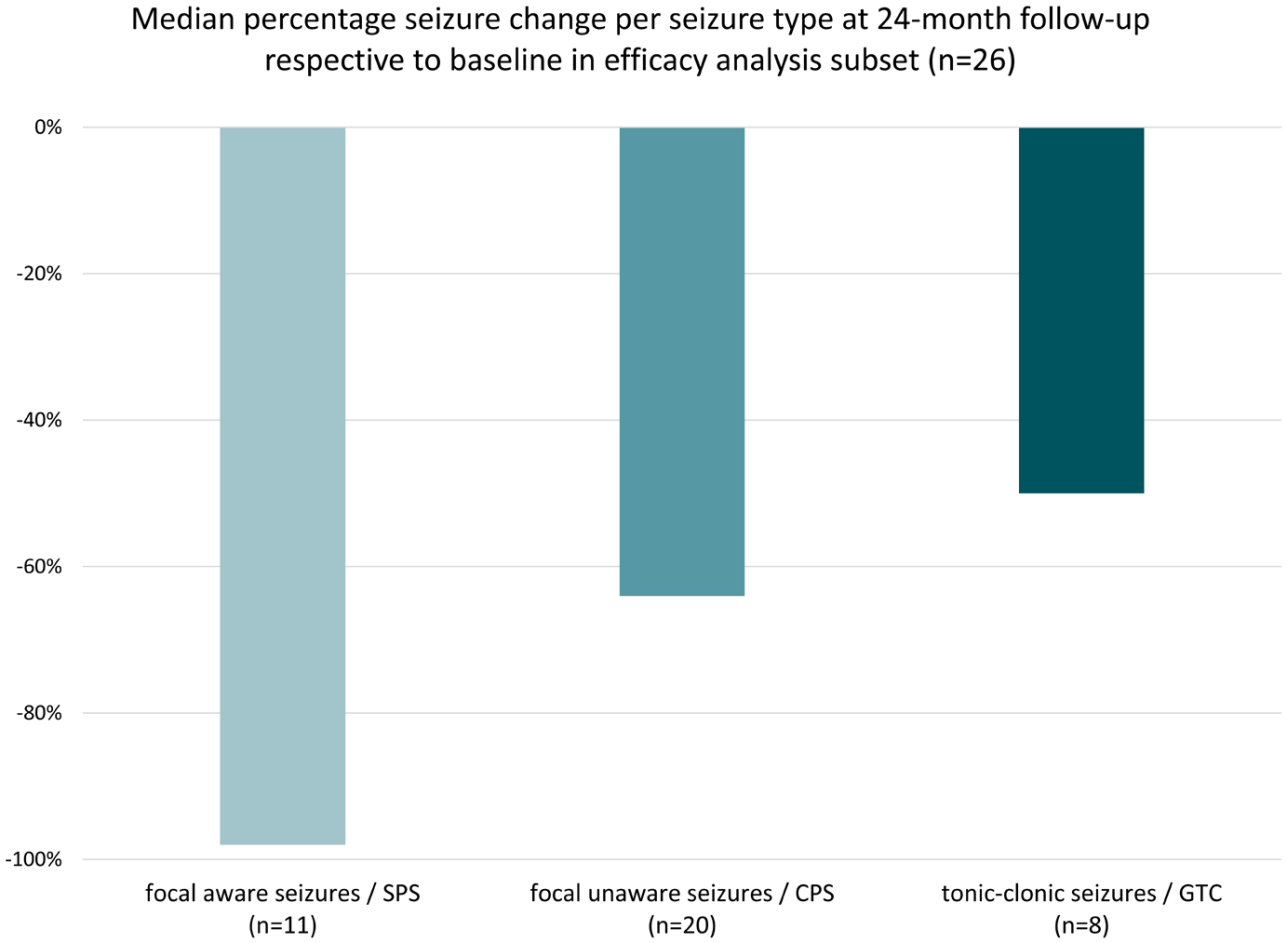
Reduction in seizure frequency per seizure type from baseline to month 23 after stimulation start. One patient with unclear seizure classification and possible nonepileptic events was excluded, as reported severe seizures could not be verified during inpatient monitoring. CPS, complex partial seizure; GTC, generalized tonic–clonic; SPS, simple partial seizure.

Treatment response was seen across the whole age spectrum of 18–75 years, and the mean age of responders was similar to that of nonresponders (responders: 35.2 ± 14.9 years, nonresponders: 35.9 ± 15.1 years). Efficacy was observed for all seizure types, namely, focal aware seizures were reduced by 98%, focal unaware seizures by 64%, and focal to bilateral tonic–clonic seizures were reduced by 50% at the last month of treatment compared to baseline (Figure 3).

Furthermore, treatment response was found across the spectrum of etiologies, with subgroups being too small for statistical analyses. The spectrum of MRI‐based assumed etiologies in individual patients with a seizure reduction by at least 90% included focal cortical dysplasia, hemangiomatosis, polymicrogyria, postencephalitic, and posttraumatic epilepsy as well as patients without MRI lesion. Overall, nonlesional patients in whom electrode placement was performed mostly EEG‐based had a similar responder rate (67%) as lesional patients (65%). Responders were found with temporal, frontal, and other localizations of stimulation.

In individual patients, stimulation parameters were adapted or stimulation mode was changed in the course of treatment by decision of the individual treating physician. Of eight patients in whom HFS was switched to low‐frequency stimulation (0.5/1 Hz), two nonresponders after 6 months of stimulation became responders at 2‐year follow‐up, and one patient improved from 80% seizure reduction to 100% seizure reduction, yet two patients who had been responders with HFS were nonresponders at 2 years.

**FIGURE 4:**
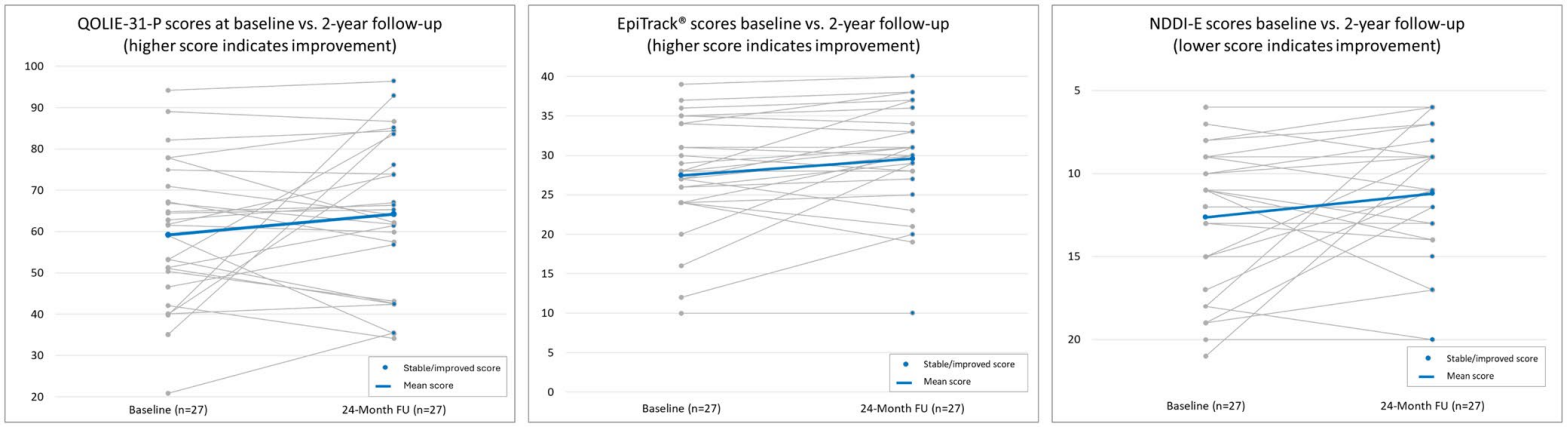
Evolution of patient‐reported outcomes between baseline and 24‐month follow‐up (FU) for the 27 patients with available data. Quality of life (QoL) as assessed with the Quality of Life in Epilepsy–Problems questionnaire (QOLIE‐31‐P) showed a significant improvement. Improvements were also found in cognitive scores as assessed by EpiTrack; however, scores were uncorrected for repeated administration. Mean Neurological Disorders Depression Inventory for Epilepsy (NDDI‐E) scores showed a minor improvement over time.

Additional patient‐reported outcomes (Figure 4) showed minor mood improvements over time, with a reduction in NDDI‐E score from 13.35 ± 1.02 at baseline to 12.63 ± 0.96 at 24‐month follow‐up. Neurocognition as assessed by EpiTrack showed a statistically significant yet minor improvement from 27.18 ± 2.12 at baseline to 29.48 ± 2.12 at 24‐month follow‐up (*p* = .007). Health‐related QoL improved significantly at 24‐month follow‐up (QOLIE‐31‐P total score 62.8 ± 4.8 vs. 58.2 ± 4.4 at baseline), with gains particularly in the subdomains seizure worries (median = +10), energy (median = +8), and daily activities (median = +7).

During the 2‐year follow‐up, changes in ASM were not uncommon. However, the mean number of ASMs taken by patients who continued treatment remained nearly unchanged, with values of 2.85 at baseline and 2.96 after 2 years. Similarly, the overall daily drug load, calculated based on the defined daily dose (DDD), showed minimal change, from 3.94 ± 1.8 at baseline to 3.83 ± 2.1 after 2 years. When analyzed by treatment response, there were no significant changes in the mean number of ASMs in either responders or nonresponders over time. However, the drug load significantly decreased in the responder group, declining from 3.99 ± 1.9 at baseline to 3.69 ± 1.9 at 2 years (*p* < 0.01), suggesting that the observed treatment response is not attributable to pharmacological adjustments. In contrast, nonresponders exhibited a nonsignificant increase in drug load, from 3.84 ± 1.7 at baseline to 4.11 ± 2.6 after 2 years.

## 4 DISCUSSION

Data from this pooled analysis of two prospective, single‐arm clinical trials confirm the excellent long‐term tolerability of epicranial FCS, as well as stable—or even improved—efficacy in patients who continued stimulation treatment. The continuation rate exceeding 80% is particularly noteworthy, given that patients were required to make an active decision to undergo generator replacement to maintain treatment during the analyzed period. This early replacement was necessary because first‐generation devices with limited battery capacity were used in the trials; these have since been replaced by newer, longer lasting models.

Increasing efficacy of neurostimulation over time has been reported in several long‐term follow‐up reports,^1^, ^22^ including for responsive neurostimulation (RNS) of the epileptic focus^23^, ^24^, ^25^, ^26^, ^27^ and for DBS of the anterior nuclei of the thalamus (ANT stimulation).^28^, ^29^, ^30^ Based on published results from other neurostimulation approaches, after 2 years of open‐label follow‐up, RNS resulted in 53% median seizure reduction,^24^ and DBS in 49% median seizure reduction in the constant SANTE cohort,^31^ whereas 31% median seizure reduction was reported in the open‐label European MORE registry of ANT stimulation.^32^ With a median seizure reduction of 68%, FCS appears to be well within the range of treatment efficacy of RNS and ANT‐DBS, even if this finding certainly needs to be verified in larger patient populations.^33^

Although responder enrichment due to decreasing participant numbers can lead to artificially elevated responder rates in long‐term trials,^34^ the FCS studies reported here demonstrate that >80% of patients continued treatment for 24 months. A similar outcome was observed in an LOCF analysis, supporting the conclusion that the observed stable or improving long‐term efficacy is not solely attributable to the continued participation of responders. Even under a worst‐case scenario—assuming all missing data represent nonresponse—the responder rate remained at 51.5%.

Seizures of all types occurred less frequently over the 2‐year treatment period. Moreover, although one seizure cluster and one episode of status epilepticus were reported during the early phase of treatment, no instances of seizure clustering or status epilepticus were reported as AEs during long‐term FCS. Notably, the percentage of patients with 75% and 90% seizure reduction also increased over time, reflecting further improvements in the intraindividual treatment response, possibly due to long‐lasting changes in network excitability.^3^, ^4^ Such major seizure reductions may have contributed to the documented significant improvements in health‐related QoL.

The absence of SAEs deemed related to the study device over the 2‐year period is noteworthy and may suggest a favorable safety profile of FCS. The extracranial placement of electrodes^35^ eliminates the risks associated with intracranial procedures, such as bleeding or infection. To date, only a single case of device site inflammation was reported following a second generator replacement surgery, which resolved with antibiotic treatment.

As with any neurostimulation technique, additional safety parameters—particularly cognitive and emotional tolerability—must be evaluated. In the present cohort, no cognitive or mood deterioration was observed, as evidenced by both objective assessments^36^ and the absence of patient‐reported complaints related to stimulation of functionally eloquent brain regions. Specifically, there were no reports of motor deficits associated with central stimulation, nor any language impairments following stimulation of the left frontal operculum or the left posterior superior temporal gyrus.

A contribution of placebo effects to the observed treatment outcomes cannot be fully excluded in this long‐term follow‐up study. This represents an inherent limitation of any long‐term outcome analysis, as the inclusion of a long‐term placebo control group is considered unethical, particularly in light of the increased risk of sudden unexpected death in epilepsy (SUDEP) observed in placebo‐treated cohorts in pharmacological trials.^37^

Another limitation of the study is that pharmacological antiseizure treatment was adjusted in a substantial subgroup of patients over the 2‐year follow‐up period, which could have contributed to improved seizure control. Although a beneficial effect of medication changes cannot be ruled out on an individual level, the mean number of ASMs among patients who continued treatment remained largely unchanged compared to baseline. Notably, in the responder group, the mean drug load—as measured by the DDD of ASMs—was lower at the end of the follow‐up than at baseline, in contrast to the nonresponder group. This finding provides preliminary evidence that improved seizure control achieved through FCS may also help reduce the pharmacological burden.

Limitations of the analyses include the moderate size of the patient cohorts. Furthermore, seizure documentation was based on paper‐and‐pencil diaries, as is presently standard in clinical trials on epilepsy treatment. This clinical standard, however, has notorious problems in validity (e.g., Schulze‐Bonhage,^14^, ^15^, ^38^) and may lead to underreporting of seizures, as encountered in other clinical trials with medical, surgical, and neuromodulatory therapies. This problem may be overcome by objective seizure assessment in the future^39^; presently, however, objective seizure documentation works reliably only for a subgroup of seizure types.^40^

Further studies will be needed to clarify the most relevant mechanisms of action contributing to the efficacy of FCS. According to the study protocol, DC‐like stimulation and HFS were performed in combination. It thus remains to be determined whether both stimulation types have additive effects or whether one of the two stimulation components exerts the major impact, and whether the application of modified stimulation parameters such as low‐frequency stimulation offers advantages. Larger patient cohorts will furthermore be needed to identify predictors of a good treatment response, which is presently addressed in a multicenter observational study.^41^ In the studies reported here, responders were found across all ages, target locations, and suspected etiologies, yet the number of patients was too small to perform valid statistical subgroup analyses.

## 5 CONCLUSIONS

Long‐term follow‐up of patients undergoing epicranial FCS shows high patient adherence and maintained treatment efficacy over time, with increasing median seizure reduction and responder rates over 2 years following implantation. Efficiency was observed in a patient cohort with a high number of failed ASMs, across different etiologies, focus localizations, and ages. Neither implantation‐ nor stimulation‐related SAEs were reported during the 2‐year follow‐up, confirming a favorable safety profile of this new neuromodulatory treatment.

## AUTHOR CONTRIBUTIONS

Andreas Schulze‐Bonhage conceptualized the article, led the clinical trial, performed data analyses, wrote the first draft of the manuscript, and edited the manuscript. Carolin Jenkner performed statistical analyses of the seizure frequencies (in total), responder rates, and adverse events. Andreas Schulze‐Bonhage performed all other statistical analyses. All authors contributed to data acquisition and to reviewing and editing the manuscript.

## CONFLICT OF INTEREST STATEMENT

A.S.‐B. has received research support from Bial, Precisis, and UNEEG, and personal honoraria for lectures or advice from Angelini Pharma, Eisai, Jazz Pharmaceuticals, Precisis, UCB, and UNEEG. M.H. has received lecture fees or grants from UCB, Angelini Pharma, and UNEEG. A.M. has received consultancy fees from LivaNova and speaker fees from Angelini Pharma. E.K. has received personal honoraria for lectures or advice from UCB, UNEEG, Medtronic, Desitin, and Precisis and has participated in trials sponsored by Medtronic, UCB, Ergomed, and Precisis. V.C. receives a collaborative grant from Brainlab. He serves as an advisor for CereGate and CorTec. He has an ongoing investigator‐initiated trial with Boston Scientific. M.G. has received honoraria and educational grants from Precisis, LivaNova, Abbott, Medtronic, Boston Scientific, and Nevro. S.G. reports research funding from patient groups, Bundesministerium für Bildung und Forschung, DFG (SPP2177 Radiomics), UM Mainz, Abbott, Boston Scientific, Böhringer Foundation, Magventure, National MS Society, Precisis, and Innovationsfond GBA (01NVF22107, INSPIRE*—*PNRM+), and lecture fees from Abbott, AbbVie, Bial, BVDN, Ipsen, Stada, and UCB. YW reports honoraria for educational presentations and consultations from Angelini Pharma, Arvelle Therapeutics, Bayer, BIAL, Bioprojet, Eisai, Idorsia Pharmaceuticals, Jazz Pharmaceuticals, LivaNova, Novartis, and UCB Pharma. None of the other authors has any conflict of interest to disclose.

## ETHICS STATEMENT

We confirm that we have read the Journal's position on issues involved in ethical publication and affirm that this report is consistent with those guidelines. The study protocols were approved by the ethics committees of all participating investigational sites and by the competent authorities in Germany (six sites) and Belgium (one site). All patients provided written consent to participate in the study. Both studies were registered in the German Clinical Trials Register (DRKS00015918, DRKS00017833), and their meta‐analysis under CRD42021266440. The data that support the findings of this study are pseudonymized only and are not publicly available on the patient level. A signed data sharing agreement is required before access to any data can be provided.

## Supporting information


File S1.

Table S1.

## Data Availability

The data that support the findings of this study are available on request from the corresponding author. The data are not publicly available due to privacy or ethical restrictions.

## References

[1] Simpson HD , Schulze‐Bonhage A , Cascino GD , Fisher RS , Jobst BC , Sperling MR , et al. Practical considerations in epilepsy neurostimulation. Epilepsia. 2022;63:2445–2460. 35700144 10.1111/epi.17329 PMC9888395

[2] Schulze‐Bonhage A , Nitsche MA , Rotter S , Focke NK , Rao VR . Neurostimulation targeting the epileptic focus: current understanding and perspectives for treatment. Seizure. 2024;117:183–192. 38452614 10.1016/j.seizure.2024.03.001

[3] Schulze‐Bonhage A , Hirsch M , Knake S , Kaufmann E , Kegele J , Rademacher M , et al. Focal cortex stimulation with a novel implantable device and antiseizure outcomes in 2 prospective multicenter single‐arm trials. JAMA Neurol. 2023;80:588–596. 37010826 10.1001/jamaneurol.2023.0066 PMC10071400

[4] Schulze‐Bonhage A . Intracranial and epicranial focus stimulation–concepts and approval status–English Version. Clin Epileptol. 2023;36:125–129.

[5] Hirsch M , Coenen VA , Schulze‐Bonhage A . Termination of seizures by ictal transcranial focal cortex stimulation. Epilepsia Open. 2023;8:673–677. 36929857 10.1002/epi4.12728 PMC10235555

[6] Liebetanz F , Klinker D , Hering R , Koch R , Nitsche MA , Potschka H , et al. Anticonvulsant effects of transcranial direct‐current stimulation (tDCS) in the rat cortical ramp model of focal epilepsy. Epilepsia. 2006;47:1216–1224. 16886986 10.1111/j.1528-1167.2006.00539.x

[7] Nelson TS , Suhr CL , Lai A , Halliday AJ , Freestone DR , McLean KJ , et al. Seizure severity and duration in the cortical stimulation model of experimental epilepsy in rats: a longitudinal study. Epilepsy Res. 2010;89:261–270. 20153951 10.1016/j.eplepsyres.2010.01.010

[8] Lu H , Gallinaro JV , Rotter S . Network remodeling induced by transcranial brain stimulation: a computational model of tDCS‐triggered cell assembly formation. Netw Neurosci. 2019;3:924–943. 31637332 10.1162/netn_a_00097 PMC6777963

[9] Ali MM , Sellers KK , Fröhlich F . Transcranial alternating current stimulation modulates large‐scale cortical network activity by network resonance. J Neurosci. 2013;33:11262–11275. 23825429 10.1523/JNEUROSCI.5867-12.2013 PMC6618612

[10] Jamil A , Batsikadze G , Kuo HI , Labruna L , Hasan A , Paulus W , et al. Systematic evaluation of the impact of stimulation intensity on neuroplastic after‐effects induced by transcranial direct current stimulation. J Physiol. 2017;595:1273–1288. 27723104 10.1113/JP272738 PMC5309387

[11] Nitsche MA , Fricke K , Henschke U , Schlitterlau A , Liebetanz D , Lang N , et al. Pharmacological modulation of cortical excitability shifts induced by transcranial direct current stimulation in humans. J Physiol. 2003;553:293–301. 12949224 10.1113/jphysiol.2003.049916 PMC2343495

[12] San‐Juan D , Morales‐Quezada L , Orozco Garduño AJ , Alonso‐Vanegas M , González‐Aragón MF , Espinoza López DA , et al. Transcranial direct current stimulation in epilepsy. Brain Stimul. 2015;8:455–464. 25697590 10.1016/j.brs.2015.01.001

[13] Vöröslakos M , Takeuchi Y , Brinyiczki K , Zombori T , Oliva A , Fernández‐Ruiz A , et al. Direct effects of transcranial electric stimulation on brain circuits in rats and humans. Nat Commun. 2018;9:483. 29396478 10.1038/s41467-018-02928-3 PMC5797140

[14] Schulze‐Bonhage A . Epicranial focal cortex stimulation with the EASEE system. In: Rao VR , editor. Neurostimulation for epilepsy. Advances, applications and opportunities. London: Academic Press; 2023a. p. 161–174.

[15] Schulze‐Bonhage A . Intracranial and epicranial focus stimulation—concepts and approval status—English version. Clin Epileptol. 2023b;36(Suppl 2):125–129. 10.1007/s10309-023-00595-z

[16] Cramer JA , Baker GA , Jacoby A . Development of a new seizure severity questionnaire: initial reliability and validity testing. Epilepsy Res. 2002;48(3):187–197. 11904237 10.1016/s0920-1211(02)00003-7

[17] Cramer JA , Perrine K , Devinsky O , Bryant‐Comstock L , Meador K , Hermann B . Development and cross‐cultural translations of a 31‐item quality of life in epilepsy inventory. Epilepsia. 1998;39:81–88. 9578017 10.1111/j.1528-1157.1998.tb01278.x

[18] Metternich B , Wagner K , Buschmann F , Anger R , Schulze‐Bonhage A . Validation of a German version of the neurological disorders depression inventory for epilepsy (NDDI‐E). Epilepsy Behav. 2012;25(4):485–488. 10.1016/j.yebeh.2012.10.00 23153711

[19] Lutz MT , Helmstaedter C . EpiTrack: tracking cognitive side effects of medication on attention and executive functions in patients with epilepsy. Epilepsy Behav. 2005;7:708–714. 16266826 10.1016/j.yebeh.2005.08.015

[20] Jacobson NS , Truax P . Clinical significance: a statistical approach to defining meaningful change in psychotherapy research. J Consult Clin Psychol. 1991;59:12–19. 2002127 10.1037//0022-006x.59.1.12

[21] Hohmann L , Bien CG , Holtkamp M , Grewe P . German questionnaires assessing quality of life and psycho‐social status in people with epilepsy: reliable change and intercorrelations. Epilepsy Behav. 2024;150:109554. 38041998 10.1016/j.yebeh.2023.109554

[22] Schulze‐Bonhage A . Long‐term outcome in neurostimulation of epilepsy. Epilepsy Behav. 2019;91:25–29. 30929666 10.1016/j.yebeh.2018.06.011

[23] Bergey GK , Morrell MJ , Mizrahi EM , Goldman A , King‐Stephens D , Nair D , et al. Long‐term treatment with responsive brain stimulation in adults with refractory partial seizures. Neurology. 2015;84:810–817. 25616485 10.1212/WNL.0000000000001280 PMC4339127

[24] Heck CN , King‐Stephens D , Massey AD , Nair DR , Jobst BC , Barkley GL , et al. Two‐year seizure reduction in adults with medically intractable partial onset epilepsy treated with responsive neurostimulation: final results of the RNS system pivotal trial. Epilepsia. 2014;55:432–441. 24621228 10.1111/epi.12534 PMC4233950

[25] Morrell MJ . Responsive cortical stimulation for the treatment of medically intractable partial epilepsy. Neurology. 2011;77:1295–1304. 21917777 10.1212/WNL.0b013e3182302056

[26] Nair DR , Laxer KD , Weber PB , Murro AM , Park YD , Barkley GL , et al. RNS system LTT study. Nine‐year prospective efficacy and safety of brain‐responsive neurostimulation for focal epilepsy. Neurology. 2020;95:e1244–e1256. 32690786 10.1212/WNL.0000000000010154 PMC7538230

[27] Roa JA , Marcuse L , Fields M , Vega‐Talbott M , Yoo JY , Wolf SM , et al. Long‐term outcomes after responsive neurostimulation for treatment of refractory epilepsy: a single‐center experience of 100 cases. J Neurosurg. 2023;139:1463–1470. 37655833 10.3171/2023.2.JNS222116

[28] Kaufmann E , Peltola J , Colon AJ , Lehtimäki K , Majtanik M , Mai JK , et al. Long‐term evaluation of anterior thalamic deep brain stimulation for epilepsy in the European MORE registry. Epilepsia. 2024;65:2438–2458. 38837755 10.1111/epi.18003

[29] Salanova V , Sperling MR , Gross RE , Irwin CP , Vollhaber JA , Giftakis JE , et al. The SANTÉ study at 10 years of follow‐up: effectiveness, safety, and sudden unexpected death in epilepsy. Epilepsia. 2021;62:1306–1317. 33830503 10.1111/epi.16895

[30] Salanova V , Witt T , Worth R , Henry TR , Gross RE , Nazzaro JM , et al. SANTE study groupLong‐term efficacy and safety of thalamic stimulation for drug‐resistant partial epilepsy. Neurology. 2015;84:1017–1025. 25663221 10.1212/WNL.0000000000001334 PMC4352097

[31] Fisher R , Salanova V , Witt T , Worth R , Henry T , Gross R , et al. Electrical stimulation of the anterior nucleus of thalamus for treatment of refractory epilepsy. Epilepsia. 2010;51:899–908. 20331461 10.1111/j.1528-1167.2010.02536.x

[32] Peltola J , Colon AJ , Pimentel J , Coenen VA , Gil‐Nagel A , Gonçalves Ferreira A , et al. Deep brain stimulation of the anterior nucleus of the thalamus in drug‐resistant epilepsy in the MORE multicenter patient registry. Neurology. 2023;100:e1852–e1865. 36927882 10.1212/WNL.0000000000206887 PMC10159763

[33] Schulze‐Bonhage A , San Antonio‐Arce V , Kalousios S , Martinez‐Lizana E , Coenen V , Hirsch M . Epicranial focal cortex stimulation for minimally invasive neuromodulation of the epileptogenic region: a review. Epilepsy Behav. 2025;168:110390. 10.1016/j.yebeh.2025.110390 40184829

[34] Ryvlin P , Rheims S , Hirsch LJ , Sokolov A , Jehi L . Neuromodulation in epilepsy: state‐of‐the‐art approved therapies. Lancet Neurol. 2021;20:1038–1047. 34710360 10.1016/S1474-4422(21)00300-8

[35] Coenen VA , Jarc N , Hirsch M , Reinacher PC , Steinhoff BJ , Bast T , et al. Technical note: preliminary surgical experience with a new implantable epicranial stimulation device for chronic focal cortex stimulation in drug‐resistant epilepsy. Acta Neurochir. 2024;166:145. 38514531 10.1007/s00701-024-06022-0 PMC10957708

[36] Wagner K , Gorny I , Kustermann J , Metternich B , Reinacher R , Coenen , et al. Cognitive outcome after epicranial cortex stimulation of the epileptic focus, in preparation. 10.1002/epi4.70216 PMC13052126 41618965

[37] Ryvlin P , Cucherat M , Rheims S . Risk of sudden unexpected death in epilepsy in patients given adjunctive antiepileptic treatment for refractory seizures: a meta‐analysis of placebo‐controlled randomised trials. Lancet Neurol. 2011;10(11):961–968. 21937278 10.1016/S1474-4422(11)70193-4

[38] Zabler N , Swinnen L , Biondi A , Novitskaya Y , Schütz E , Epitashvili N , et al. High precision in epileptic seizure self‐reporting with an app diary. Sci Rep. 2024;14:15823. 38982283 10.1038/s41598-024-66932-y PMC11233562

[39] Rocamora R , Baumgartner C , Novitskaya Y , Hirsch M , Koren J , Villla L , et al. The spectrum of indications for ultralong‐term EEG monitoring. Seizure. 2024;121:262–270. 39326109 10.1016/j.seizure.2024.08.015

[40] Beniczky S , Wiebe S , Jeppesen J , Tatum WO , Brazdil M , Wang Y , et al. Automated seizure detection using wearable devices: a clinical practice guideline of the international league against epilepsy and the International Federation of Clinical Neurophysiology. Clin Neurophysiol. 2021;132:1173–1184. 33678577 10.1016/j.clinph.2020.12.009

[41] Kalousios S , Hesser J , Dümpelmann M , Baumgartner C , Hamer HM , Hirsch M , et al. Therapy response prediction of focal cortex stimulation based on clinical parameters: a multicentre, non‐interventional study protocol. BMJ Open. 2025;15:e089903. 10.1136/bmjopen-2024-089903 PMC11873335 39956604

