# Supplementary material for: Two‐year outcomes of epicranial focal cortex stimulation in pharmacoresistant focal epilepsy
Source: Epilepsia. 2025 May 16;66(9):3242–53. doi: 10.1111/epi.18448 (PMC12455469; doi:10.1111/epi.18448)
Supplement: Supplementary file 1 — File S1. [file EPI-66-3242-s002.pdf]

Supplementary file 1.

List of Inclusion- and Exclusion criteria
to the clinical trials EASEE-II and PIMIDES-I

A. Inclusion Criteria

Patients enrolled in the study must meet all of the following criteria:

1. Patients with a clinical diagnosis of focal seizures or focal to bilateral tonic clonic seizures.
2. Patients with a diagnosis of lateral temporal lobe epilepsy or extra-temporal lobe epilepsy.
3. Patients with a predominant epileptic focus, which can be clearly identified as the site of implantation for the electrode based on EEG and clinical presentation.
4. (PIMIDES only) Patients who are able to initiate a stimulation bolus during their seizure (intact consciousness at least during part of a habitual seizure type, as well as motor ability to operate the device to trigger stimulation in this phase).
5. Patients, if they have had prior resective surgery to treat epilepsy, need to have a clearly identifiable epileptic focus and a preserved neocortex in the region of implantation.
6. Patients who have failed treatment with a minimum of two anti-seizure medications (used in appropriate doses).
7. Patients having seizures which are distinct, stereotypical events and can be reliably counted, in the opinion of the Investigator, by the patient or caregiver and recorded in a seizure diary.
8. Patients having an anticipated average of 3-200 partial-onset seizures (focal to bilateral tonic clonic seizures) during the baseline period.
9. Patients taking a constant dose of antiepileptic medication(s) over the most recent 28-day period prior to the baseline period (use of medication for acute treatment of seizures is allowed).
10. Patients between the ages of 18 and 75 years.
11. Patients able and willing to provide appropriate consent prior to study procedures.
12. Patients able to complete regular office appointments per the protocol requirements, including behavioral (mood) surveys and neuropsychological testing.
13. Patients willing to be implanted with the EASEE® System as a treatment for his/her seizures.

B. Exclusion Criteria

Patients who meet any of the following criteria are not eligible to be enrolled in the study:

1. Patients with a diagnosis of mesial temporal lobe epilepsy.
2. Patients with a previous diagnosis of psychogenic or non-epileptic seizures, which are semiologically non-distinguishable from epileptic seizures.
3. Patients with a diagnosis of primarily generalized seizures.
4. Patients, if after resective surgery, with non-preserved neocortex in the region of implantation
5. Patients with unprovoked status epilepticus in the preceding 6 months prior to enrolment.
6. Patients with a clinically significant or unstable medical condition (including cardiac conditions, alcohol and/or drug abuse) or a progressive central nervous system disease
7. Patients with a diagnosis of active psychosis, major depression or suicidal ideation in the preceding year (excluding postictal psychosis).
8. Females who are pregnant or have a pregnancy wish in the next 2 years.
9. Patients enrolled in a therapeutic investigational drug or device trial.
10. Patients who are anatomically not eligible for EASEE® System implant in the opinion of the Investigator.
11. Patients with an implanted electronic medical device that delivers electrical energy to the body (e.g. DBS, cardiac pacemaker or defibrillator) except for an existing VNS device that can be reliably switched off for the duration of the trial.
12. Patients requiring scheduled MRIs during the study phase.
13. (PIMIDES only)Patients who are unable to properly operate the EASEE® Access handheld device (no intact consciousness during part of habitual seizure type or no motor ability to operate device for bolus stimulation).
