# Supplementary material for: Two‐year outcomes of epicranial focal cortex stimulation in pharmacoresistant focal epilepsy
Source: Epilepsia. 2025 May 16;66(9):3242–53. doi: 10.1111/epi.18448 (PMC12455469; doi:10.1111/epi.18448)
Supplement: Supplementary file 2 — Table S1. [file EPI-66-3242-s001.docx]

# Supplementary Information

Reported percentage of patients with a given adverse event during the 24-month period following device implantation (cut-off >5%)

| ***Incidence of AEs by System Organ Class (SOC), and Preferred Term (PT)*** | | | |
| --- | --- | --- | --- |
|  | | ***Adverse Events Incidence*** | |
| ***System Organ Class*** | ***Preferred term*** | ***No.*** | ***Total %*** |
| Total number of patients |  | 33 | 100.0 |
| Number of patients with at least one AE |  | 27 | 81.8 |
| Nervous system disorders |  | 21 | 63.6 |
|  | Headache | 11 | 33.3 |
|  | Epilepsy | 10 | 30.3 |
|  | Dizziness | 5 | 15.2 |
|  | Seizure | 5 | 15.2 |
|  | Change in seizure presentation | 2 | 6.1 |
|  | Paraesthesia | 2 | 6.1 |
|  | Status epilepticus | 2 | 6.1 |
| General disorders and administration site conditions |  | 13 | 39.4 |
|  | Fatigue | 4 | 12.1 |
|  | Medical device site pain | 3 | 9.1 |
|  | Implant site pain | 2 | 6.1 |
| Infections and infestations |  | 10 | 30.3 |
|  | COVID-19 | 3 | 9.1 |
|  | Coronavirus infection | 2 | 6.1 |
|  | Nasopharyngitis | 2 | 6.1 |
|  | Urinary tract infection | 2 | 6.1 |
| Injury, poisoning and procedural complications |  | 10 | 30.3 |
|  | Contusion | 2 | 6.1 |
|  | Fall | 2 | 6.1 |
|  | Head injury | 2 | 6.1 |
|  | Procedural pain | 2 | 6.1 |
|  | Toxicity to various agents | 2 | 6.1 |
| Musculoskeletal and connective tissue disorders |  | 7 | 21.2 |
|  | Pain in extremity | 3 | 9.1 |
| Gastrointestinal disorders |  | 6 | 18.2 |
|  | Nausea | 2 | 6.1 |
| Psychiatric disorders |  | 5 | 15.2 |
|  | Depressive symptom | 2 | 6.1 |
| Investigations |  | 4 | 12.1 |
| Eye disorders |  | 3 | 9.1 |
| Respiratory, thoracic and mediastinal disorders |  | 3 | 9.1 |
| Vascular disorders |  | 3 | 9.1 |
|  | Haematoma | 2 | 6.1 |
| Ear and labyrinth disorders |  | 2 | 6.1 |
|  |  |  |  |
